# Supplementary material for: Modeling glioblastoma heterogeneity as a dynamic network of cell states
Source: Mol Syst Biol. 2021 Sep 16;17(9):e10105. doi: 10.15252/msb.202010105 (PMC8444284; doi:10.15252/msb.202010105)
Supplement: Supplementary file 6 — Source Data for Figure 5 [file MSB-17-e10105-s004.zip › Figure5A_sourcedata/GSEA_3071/nosigdb_stateI.GseaPreranked.1622795145664/FELDMAN_2020_S.html]

Details for gene set FELDMAN\_2020\_S[GSEA]

|  || Dataset | state13071 |
| Phenotype | NoPhenotypeAvailable |
| Upregulated in class | na\_neg |
| GeneSet | FELDMAN\_2020\_S |
| Enrichment Score (ES) | -0.59380513 |
| Normalized Enrichment Score (NES) | -3.2079132 |
| Nominal p-value | 0.0 |
| FDR q-value | 0.0 |
| FWER p-Value | 0.0 |
Table: GSEA Results Summary

  

Fig 1: Enrichment plot: FELDMAN\_2020\_S      
 Profile of the Running ES Score & Positions of GeneSet Members on the Rank Ordered List

  

| PROBE | GENE SYMBOL | GENE\_TITLE | RANK IN GENE LIST | RANK METRIC SCORE | RUNNING ES | CORE ENRICHMENT || 1 | GJB2 |  |  | 8 | 1.906 | 0.0203 | No |
| 2 | SLC20A1 |  |  | 116 | 1.035 | 0.0011 | No |
| 3 | CALD1 |  |  | 201 | 0.824 | -0.0139 | No |
| 4 | TPM1 |  |  | 419 | 0.575 | -0.0711 | No |
| 5 | LIMS1 |  |  | 498 | 0.514 | -0.0880 | No |
| 6 | FTL |  |  | 564 | 0.473 | -0.1016 | No |
| 7 | BAMBI |  |  | 740 | 0.400 | -0.1484 | No |
| 8 | ARL6IP5 |  |  | 756 | 0.395 | -0.1482 | No |
| 9 | DDIT4 |  |  | 865 | 0.361 | -0.1757 | No |
| 10 | MALAT1 |  |  | 1252 | 0.276 | -0.2863 | No |
| 11 | PHLDA1 |  |  | 1262 | 0.275 | -0.2857 | No |
| 12 | CCDC34 |  |  | 1376 | 0.255 | -0.3160 | No |
| 13 | MSH2 |  |  | 1438 | -0.252 | -0.3310 | No |
| 14 | ARL6IP6 |  |  | 1577 | -0.265 | -0.3686 | No |
| 15 | HSPA8 |  |  | 1605 | -0.268 | -0.3734 | No |
| 16 | HNRNPF |  |  | 1636 | -0.272 | -0.3790 | No |
| 17 | SMC3 |  |  | 1650 | -0.274 | -0.3796 | No |
| 18 | CCNB2 |  |  | 1757 | -0.288 | -0.4074 | No |
| 19 | EXOSC9 |  |  | 1777 | -0.291 | -0.4096 | No |
| 20 | RAD51 |  |  | 1845 | -0.299 | -0.4258 | No |
| 21 | RAN |  |  | 1907 | -0.306 | -0.4402 | No |
| 22 | TGIF1 |  |  | 1955 | -0.313 | -0.4503 | No |
| 23 | SNRPB |  |  | 2064 | -0.328 | -0.4783 | No |
| 24 | EIF1AX |  |  | 2103 | -0.335 | -0.4855 | No |
| 25 | CENPA |  |  | 2220 | -0.357 | -0.5155 | No |
| 26 | MSH6 |  |  | 2362 | -0.386 | -0.5525 | No |
| 27 | AURKA |  |  | 2467 | -0.404 | -0.5784 | No |
| 28 | HAUS8 |  |  | 2494 | -0.412 | -0.5811 | No |
| 29 | CACYBP |  |  | 2536 | -0.424 | -0.5882 | No |
| 30 | TEX30 |  |  | 2556 | -0.428 | -0.5887 | Yes |
| 31 | H2AFZ |  |  | 2572 | -0.433 | -0.5880 | Yes |
| 32 | RPL22L1 |  |  | 2576 | -0.434 | -0.5837 | Yes |
| 33 | BEX4 |  |  | 2584 | -0.435 | -0.5806 | Yes |
| 34 | ANP32B |  |  | 2593 | -0.438 | -0.5778 | Yes |
| 35 | GGCT |  |  | 2610 | -0.442 | -0.5772 | Yes |
| 36 | SRSF7 |  |  | 2619 | -0.445 | -0.5743 | Yes |
| 37 | PPIF |  |  | 2624 | -0.446 | -0.5702 | Yes |
| 38 | EIF2S1 |  |  | 2638 | -0.450 | -0.5686 | Yes |
| 39 | DUT |  |  | 2640 | -0.450 | -0.5636 | Yes |
| 40 | DCTPP1 |  |  | 2644 | -0.451 | -0.5591 | Yes |
| 41 | EXOSC8 |  |  | 2659 | -0.456 | -0.5578 | Yes |
| 42 | PGP |  |  | 2681 | -0.468 | -0.5584 | Yes |
| 43 | MCM6 |  |  | 2699 | -0.471 | -0.5578 | Yes |
| 44 | SNRPD1 |  |  | 2701 | -0.471 | -0.5525 | Yes |
| 45 | HAT1 |  |  | 2703 | -0.472 | -0.5472 | Yes |
| 46 | YWHAH |  |  | 2725 | -0.479 | -0.5477 | Yes |
| 47 | RPA3 |  |  | 2730 | -0.480 | -0.5432 | Yes |
| 48 | UNG |  |  | 2758 | -0.489 | -0.5453 | Yes |
| 49 | BRCA2 |  |  | 2772 | -0.495 | -0.5433 | Yes |
| 50 | SRSF2 |  |  | 2782 | -0.497 | -0.5400 | Yes |
| 51 | SLBP |  |  | 2792 | -0.499 | -0.5367 | Yes |
| 52 | HNRNPAB |  |  | 2816 | -0.508 | -0.5375 | Yes |
| 53 | SIVA1 |  |  | 2822 | -0.510 | -0.5329 | Yes |
| 54 | ARL6IP1 |  |  | 2823 | -0.510 | -0.5268 | Yes |
| 55 | ASF1B |  |  | 2824 | -0.510 | -0.5207 | Yes |
| 56 | CDCA3 |  |  | 2829 | -0.513 | -0.5158 | Yes |
| 57 | TMEM97 |  |  | 2834 | -0.514 | -0.5109 | Yes |
| 58 | POLR3K |  |  | 2872 | -0.528 | -0.5155 | Yes |
| 59 | PTTG1 |  |  | 2895 | -0.540 | -0.5156 | Yes |
| 60 | ATAD5 |  |  | 2935 | -0.563 | -0.5204 | Yes |
| 61 | MIS18A |  |  | 2947 | -0.570 | -0.5169 | Yes |
| 62 | ZFAND6 |  |  | 2966 | -0.581 | -0.5153 | Yes |
| 63 | CTNNAL1 |  |  | 2976 | -0.585 | -0.5109 | Yes |
| 64 | VRK1 |  |  | 2983 | -0.592 | -0.5057 | Yes |
| 65 | SMC2 |  |  | 2984 | -0.592 | -0.4986 | Yes |
| 66 | C19orf48 |  |  | 3013 | -0.608 | -0.4997 | Yes |
| 67 | TFDP1 |  |  | 3027 | -0.617 | -0.4962 | Yes |
| 68 | DNMT1 |  |  | 3035 | -0.622 | -0.4908 | Yes |
| 69 | NOVA1 |  |  | 3040 | -0.627 | -0.4845 | Yes |
| 70 | CCND1 |  |  | 3072 | -0.655 | -0.4859 | Yes |
| 71 | CDC45 |  |  | 3081 | -0.661 | -0.4804 | Yes |
| 72 | CDCA4 |  |  | 3084 | -0.663 | -0.4731 | Yes |
| 73 | MAD2L1 |  |  | 3099 | -0.675 | -0.4692 | Yes |
| 74 | NASP |  |  | 3106 | -0.682 | -0.4628 | Yes |
| 75 | RFC3 |  |  | 3108 | -0.683 | -0.4550 | Yes |
| 76 | PA2G4 |  |  | 3116 | -0.690 | -0.4489 | Yes |
| 77 | LMNB1 |  |  | 3121 | -0.692 | -0.4418 | Yes |
| 78 | POLD3 |  |  | 3122 | -0.695 | -0.4335 | Yes |
| 79 | CENPK |  |  | 3127 | -0.700 | -0.4264 | Yes |
| 80 | RFC4 |  |  | 3129 | -0.701 | -0.4184 | Yes |
| 81 | MCM5 |  |  | 3132 | -0.706 | -0.4105 | Yes |
| 82 | GMNN |  |  | 3140 | -0.719 | -0.4041 | Yes |
| 83 | CDK1 |  |  | 3149 | -0.727 | -0.3978 | Yes |
| 84 | ORC6 |  |  | 3159 | -0.738 | -0.3916 | Yes |
| 85 | TK1 |  |  | 3162 | -0.745 | -0.3834 | Yes |
| 86 | DNAJC9 |  |  | 3167 | -0.747 | -0.3757 | Yes |
| 87 | MCM3 |  |  | 3182 | -0.761 | -0.3707 | Yes |
| 88 | USP1 |  |  | 3183 | -0.762 | -0.3617 | Yes |
| 89 | BRCA1 |  |  | 3185 | -0.765 | -0.3529 | Yes |
| 90 | PSMC3IP |  |  | 3194 | -0.772 | -0.3460 | Yes |
| 91 | CDCA7 |  |  | 3203 | -0.785 | -0.3391 | Yes |
| 92 | CDC6 |  |  | 3204 | -0.785 | -0.3297 | Yes |
| 93 | FBXO5 |  |  | 3212 | -0.797 | -0.3223 | Yes |
| 94 | CARHSP1 |  |  | 3214 | -0.801 | -0.3131 | Yes |
| 95 | RFC2 |  |  | 3245 | -0.839 | -0.3119 | Yes |
| 96 | CENPM |  |  | 3269 | -0.881 | -0.3082 | Yes |
| 97 | CHAF1A |  |  | 3270 | -0.882 | -0.2978 | Yes |
| 98 | CCNE2 |  |  | 3276 | -0.889 | -0.2886 | Yes |
| 99 | FEN1 |  |  | 3278 | -0.890 | -0.2784 | Yes |
| 100 | HELLS |  |  | 3284 | -0.901 | -0.2691 | Yes |
| 101 | RANBP1 |  |  | 3290 | -0.911 | -0.2597 | Yes |
| 102 | ZWINT |  |  | 3296 | -0.925 | -0.2502 | Yes |
| 103 | MEST |  |  | 3307 | -0.940 | -0.2420 | Yes |
| 104 | PCNA |  |  | 3319 | -0.963 | -0.2338 | Yes |
| 105 | UBE2T |  |  | 3330 | -0.979 | -0.2251 | Yes |
| 106 | MCM4 |  |  | 3341 | -1.006 | -0.2160 | Yes |
| 107 | DEK |  |  | 3344 | -1.025 | -0.2044 | Yes |
| 108 | MCM10 |  |  | 3356 | -1.050 | -0.1952 | Yes |
| 109 | ESCO2 |  |  | 3367 | -1.088 | -0.1852 | Yes |
| 110 | MCM7 |  |  | 3384 | -1.147 | -0.1763 | Yes |
| 111 | GINS2 |  |  | 3386 | -1.156 | -0.1628 | Yes |
| 112 | DHFR |  |  | 3387 | -1.159 | -0.1490 | Yes |
| 113 | HMGB2 |  |  | 3390 | -1.184 | -0.1355 | Yes |
| 114 | CLSPN |  |  | 3391 | -1.184 | -0.1214 | Yes |
| 115 | CENPU |  |  | 3398 | -1.230 | -0.1086 | Yes |
| 116 | FAM111B |  |  | 3402 | -1.238 | -0.0947 | Yes |
| 117 | LYPD1 |  |  | 3412 | -1.270 | -0.0823 | Yes |
| 118 | ATAD2 |  |  | 3415 | -1.278 | -0.0677 | Yes |
| 119 | RRM2 |  |  | 3420 | -1.297 | -0.0534 | Yes |
| 120 | TYMS |  |  | 3436 | -1.391 | -0.0413 | Yes |
| 121 | AKAP12 |  |  | 3444 | -1.501 | -0.0255 | Yes |
| 122 | SCRG1 |  |  | 3480 | -1.876 | -0.0135 | Yes |
| 123 | CDCA7L |  |  | 3481 | -1.878 | 0.0089 | Yes |
Table: GSEA details [plain text format]

  

Fig 2: FELDMAN\_2020\_S: Random ES distribution      
 Gene set null distribution of ES for **FELDMAN\_2020\_S**
